# Cerebrospinal Fluid Cell-Free DNA-Based Detection of High Level of Genomic Instability Is Associated With Poor Prognosis in NSCLC Patients With Leptomeningeal Metastases

**DOI:** 10.3389/fonc.2022.664420

**Published:** 2022-04-28

**Authors:** Xi Wu, Puyuan Xing, Min Shi, Weihua Guo, Fangping Zhao, Honglin Zhu, Jianping Xiao, Jinghai Wan, Junling Li

**Affiliations:** ^1^ General Department, National Cancer Center/National Clinical Research Center for Cancer/Cancer Hospital, Chinese Academy of Medical Sciences and Peking Union Medical College, Beijing, China; ^2^ Department of Medical Oncology, National Cancer Center/National Clinical Research Center for Cancer/Cancer Hospital, Chinese Academy of Medical Sciences and Peking Union Medical College, Beijing, China; ^3^ Hangzhou Jichenjunchuang Medical Laboratory Co., Ltd., Hangzhou, China; ^4^ Department of Radiotherapy, National Cancer Center/National Clinical Research Center for Cancer/Cancer Hospital, Chinese Academy of Medical Sciences and Peking Union Medical College, Beijing, China; ^5^ Department of Neurosurgery, National Cancer Center/National Clinical Research Center for Cancer/Cancer Hospital, Chinese Academy of Medical Sciences and Peking Union Medical College, Beijing, China

**Keywords:** cerebrospinal fluid cfDNA, leptomeningeal metastases, EGFR-TKI resistance, genomic instability, NSCLC

## Abstract

**Introduction:**

Leptomeningeal metastasis (LM) commonly occurs in non-small cell lung cancer (NSCLC) patients and has a poor prognosis. Due to limited access to leptomeningeal lesions, the genetic characteristics of LM have not been explored to date. Cerebrospinal fluid (CSF) may be the most representative liquid biopsy medium to obtain genomic information from LM in NSCLC.

**Methods:**

CSF biopsies and matched peripheral blood biopsies were collected from 33 NSCLC patients with LM. We profiled genetic alterations from LM by comparing CSF cell-free DNA (cfDNA) with plasma cfDNA. Somatic mutations were examined using targeted sequencing. Genomic instability was analyzed by low-coverage whole-genome sequencing (WGS).

**Results:**

Driver mutations were detected in 100% of CSF cfDNA with much higher variant allele frequency than that in matched plasma cfDNA (57.5%). Furthermore, we found that the proportions of CSF cfDNA fragments below 150 bp were significantly higher than those in plasma cfDNA. These findings indicate enrichment of circulating tumor DNA (ctDNA) in CSF and explain the high sensitivity of mutation detection in the CSF. The absence of some mutations in CSF cfDNA—especially the first-/second-generation mutation T790M, which confers resistance to epidermal growth factor receptor (EGFR)-Tyrosine kinase inhibitors (TKIs)—that were present in plasma cfDNA samples indicates different mechanisms of cancer evolution between LM and extracranial lesions. In addition, 86.6% of CSF ctDNA samples revealed high levels of genomic instability compared with 2.5% in plasma cfDNA samples. A higher number of large-scale state transitions (LSTs) in CSF cfDNA were associated with a shorter overall survival (OS).

**Conclusion:**

Our results suggest that LM and extracranial lesions develop independently. Both CSF cfDNA genetic profiling and plasma cfDNA genetic profiling are necessary for clinical decision-making for NSCLC patients with LM. Through CSF-based low-coverage WGS, a high level of LSTs was identified as a potential biomarker of poor prognosis.

## Introduction

Leptomeningeal metastases (LMs) occur in 3.4%–3.8% of non-small cell lung cancer (NSCLC) patients and are more common (9.4%) in patients whose tumors carry epidermal growth factor receptor (EGFR) mutations, even during EGFR-TKI treatment (1–3). Despite extensive research on extracranial lesions, the role of acquired resistance to EGFR-TKIs in LM has not been well studied due to limited access to leptomeningeal lesions. The prognosis of LM in NSCLC is very poor, with a median overall survival (OS) of 1.9 months if patients are untreated and 3.5–12 months upon treatment with EGFR-TKIs (4). Brain imaging technology (MRI) and cerebrospinal fluid (CSF) cytology are widely used in the diagnosis of LM. Despite CSF cytology being the gold standard, a positive result for this diagnostic method is observed in only 50%–60% of patients with LM at the first CSF examination (5, 6). Early diagnosis and a comprehensive understanding of genetic alterations are essential for accurate assessment of disease progression and a rational exploration of clinical treatments.

With the rapid progress in the application of liquid biopsy in the past few years, cell-free DNA (cfDNA), especially plasma cfDNA, has been used widely for monitoring tumor progression/regression and response to treatments (7, 8). However, plasma cfDNA is not effective in detecting mutations in brain tumors and brain metastasis (9–13). CSF cfDNA-based profiling of tumor-related genetic alterations has also been investigated, with some limitations, depending on the location and stage of tumors, and has shown great advantages in the case of LM (9, 11–14).

Previous studies have shown that CSF-derived cfDNA demonstrates higher sensitivity and better represents the genetic status for LM than plasma-derived cfDNA (11, 13, 15, 16). However, a fundamental understanding of LM genetics remains elusive. In this study, we focused on identifying genetic alterations (both mutations and structural variants) at both gene and genome levels in CSF cfDNA derived from NSCLC patients with LM in order to investigate the molecular mechanism of LMs and identify genetic signatures for improved clinical management.

## Materials and Methods

### Patient Enrollment

This study was conducted in accordance with the Declaration of Helsinki and approved by the Peking University Cancer Hospital Ethics Committee. All patients provided written informed consent for treatment, sample collection, and analysis. Thirty-three lung adenocarcinoma patients with LM were enrolled from March 2017 to August 2019 at the Cancer Hospital Chinese Academy of Medical Sciences. The diagnostic criteria for LM were based on a positive result on brain MRI or CSF cytologic examination. All patients received care at the Cancer Hospital Chinese Academy of Medical Sciences and had been followed for more than 6 months if death did not occur earlier.

### Cell-Free DNA Extraction

CSF cfDNA and plasma cfDNA were extracted using QIAamp Circulating Nucleic Acid Kit (Qiagen, USA) in accordance with the manufacturer’s instructions. Germline DNA was extracted from the buffy coat of the matched whole-blood samples using a DNeasy Blood and Tissue Kit (Qiagen, USA) and analyzed as a germline reference. DNA was quantified using the Qubit Fluorometer (Invitrogen, USA). Size distribution of cfDNA was determined by low-coverage whole-genome sequencing (WGS), as previously described (17).

### Mutation Profiling

Genomic bar-coded DNA libraries were constructed with 20 ng cfDNA or 500 ng genomic DNA using KAPA HyperPrep Kits (Roche, Germany) and captured using probes from three designed lung cancer panels, which covered exons of 137 genes (Geneseeq, China), 520 genes (Burning Rock Dx, China), or 180 genes (Genetron Health, China). High-throughput sequencing was performed on a HiSeq platform (Illumina, USA). Sequencing reads were mapped to a human reference genome (hg19) using the Burrows–Wheeler Aligner (BWA) (18). Duplicate removal, local realignment, and base quality recalibration were performed using PICARD (http://broadinstitute.github.io/picard/) and the Genome Analysis Toolkit (GATK). Mutations with allelic fractions of less than 0.1%, supported by fewer than 4 unique reads or detected as germline mutations, were disregarded. Mutations in 84 genes, which overlapped between the three lung cancer panels mentioned above, were analyzed in this study (**Table S1**).

### Structural Variant Analysis

Copy number variants (CNVs) from targeted sequencing data were called using copy number segments produced by ABSOLUTE v.1.4 and analyzed by GISTIC 2.0 (19).

Low-coverage WGS (3×) was performed using genomic bar-coded DNA libraries, which were constructed with KAPA HyperPrep Kits [Genetron Health (Beijing) Co. Ltd.]. PE150 reads were generated using the HiSeq 4000 platform (Illumina, USA) and aligned to the reference human genome hg19.

For a general assessment of genome instability, read counts from the WGS bam files were binned into contiguous 1-Mb windows (sex chromosomes were excluded) and GC bias was corrected using GATK. Stable chromosomal regions were identified if the coverage of each bin in the region was relatively stable and the variant allele fraction (VAF) of heterozygotic germline Single nucleotide polymorphisms (SNPs) in the region remained at about 0.5 using sequencing data from 180-gene lung cancer panel data as a reference. Coverage of each bin was adjusted to the mean coverage of stable chromosomal regions for each sample. For plasma and CSF samples, the coverage in each genomic bin was further divided by the coverage in the corresponding bin of germline DNA from the same patient. The resulting ratios were log2-transformed, and segmentation was performed using the R package DNAcopy (20).

Genomic instability was evaluated based on a number of large-scale state transitions (LSTs) and the genome instability number (GIN). Using low-coverage WGS data, the number of LSTs was quantified as the number of chromosomal breakpoints (change in coverage) between adjacent regions, each of at least 10 megabases (Mb) obtained after smoothing (filtering <3 Mb small-scale copy number variation) (21). Similarly, GIN was quantified as the sum, across all autosomal bins, of the absolute deviation of the normalized genomic representation of a sample to the expected normalized genomic representation of stable chromosome regions of the same sample (22).

### Data Availability

The mutation profiling data and raw whole-genome sequence data referenced in this study are available from The National Omics Data Encyclopedia (https://www.biosino.org/node/) under accession code OEP003149 and is available on request.

## Results

### Patient Information

All diagnoses of LMs were confirmed based on MRI or CSF cytology. Seventeen of 33 patients (51.5%) were men, and the median age of the cohort was 55 years (range, 39–78 years). Driver mutations of primary tumors were determined by qPCR or targeted sequencing: 31 out of 33 primary tumors harbored EGFR mutations (93.9%), 1 primary tumor carried Anaplastic Lymphoma kinase (ALK) rearrangement, and 1 primary tumor carried erb-b2 receptor tyrosine kinase 2 (ERBB2) mutations (**Table 1**). Most patients (31 out of 33) with EGFR alterations had a history of targeted therapies and were switched to a different EGFR-TKI upon the development of resistance (**Table 1**). Ten out of 33 patients showed no brain parenchymal metastases at the time of LM diagnosis; among them, 2 patients showed no extracranial metastases. For genomic profiling, 10 ml of CSF was collected by lumbar puncture and 10 ml of matched peripheral blood was collected by venipuncture, after LM diagnosis, from all 33 patients.

**Table 1:** Clinical information.

Pt NO.	Age at LM, years	Sex	The driver mutations in primary tumor	ECOG PS at LM	Extracranial metastases status	Brain parenchymal metastases status	TKI before CSF collection	OS from LM, months	Genomic profiling test
P01	48	man	EGFR.G719C	2	stable	stable	Icotinib+Erlotinib	16	180 genes panel and WGS
P02	46	man	EGFR.L858R	4	progressive	progressive	Icotinib+Osimertinib	2	180 genes panel and WGS
P03	56	man	EML4-ALK fusion	2	stable	without metastases	Crizotinib+Brigatinib	3	137 genes panel
P04	54	man	EGFR.exon19del	1	stable	without metastases	Gefitinib+Osimertinib+ Erlotinib	16	180 genes panel and WGS
P05	47	man	EGFR.exon19del	1	stable	without metastases	Icotinib	>14	180 genes panel and WGS
P06	62	woman	EGFR.L858R	3	stable	stable	Erlotinib+Osimertinib	12	520 genes panel
P07	75	man	EGFR.L858R	3	stable	without metastases	Erlotinib	11	137 genes panel
P08	49	woman	EGFR.exon19del	2	progressive	regressive	Gefitinib+Osimertinib+ Capmatinib	8	180 genes panel and WGS
P09	45	woman	EGFR.exon19del	2	stable	progressive	Gefitinib	>11	180 genes panel and WGS
P10	57	man	EGFR.exon19del	3	stable	without metastases	Gefitinib+Osimertinib	>13	180 genes panel and WGS
P11	48	woman	EGFR.exon19del	4	stable	progressive	Gefitinib+Osimertinib	15	180 genes panel and WGS
P12	47	woman	EGFR.L858R	3	without metastases	without metastases	Icotinib+Osimertinib+ Erlotinib+Cabozantinib	>19	180 genes panel and WGS
P13	57	woman	ERBB2.G776delinsVV	3	stable	progressive	Afatinib	6	520 genes panel
P14	58	woman	EGFR.exon19del	2	progressive	progressive	Gefitinib+Erlotinib	1	180 genes panel and WGS
P15	48	man	EGFR.L858R	4	progressive	stable	Erlotinib+Osimertinib	7	520 genes panel
P16	56	woman	EGFR.exon19del	1	progressive	without metastases	Gefitinib	>13	180 genes panel and WGS
P17	52	woman	EGFR.exon19del	2	stable	stable	Gefitinib+Osimertinib	>14	180 genes panel and WGS
P18	60	man	EGFR.L858R	3	progressive	stable	Icotinib+Erlotinib+ Osimertinib	3	180 genes panel and WGS
P19	53	man	EGFR.L858R	2	without metastases	progressive	Icotinib	6	137 genes panel
P20	39	man	EGFR.exon19del	2	regressive	without metastases	Gefitinib+Afatinib	>14	180 genes panel and WGS
P21	66	woman	EGFR.L858R	4	progressive	stable	Gefitinib+Osimertinib	0.5	180 genes panel and WGS
P22	60	woman	EGFR.L858R	1	stable	progressive	Gefitinib+Erlotinib+ Osimertinib	8	520 genes panel
P23	60	man	EGFR.L858R	3	progressive	progressive	Gefitinib	9	520 genes panel
P24	62	woman	EGFR.exon19del	2	progressive	without metastases	Gefitinib+Erlotinib	9	137 genes panel
P25	55	man	EGFR.exon19del	1	stable	progressive	Gefitinib+Erlotinib+ Osimertinib	8	180 genes panel and WGS
P26	50	woman	EGFR.L858R	2	stable	stable	Osimertinib	>12	180 genes panel and WGS
P27	64	woman	EGFR.exon19del	2	stable	progressive	Erlotinib	>11	180 genes panel and WGS
P28	79	man	EGFR.L858R	2	stable	without metastases	Icotinib	>9	180 genes panel and WGS
P29	50	woman	EGFR.exon19del	2	progressive	progressive	–	11	180 genes panel and WGS
P30	47	man	EGFR.exon19ins	1	progressive	progressive	Gefitinib+Afatinib+ Osimertinib	5	180 genes panel and WGS
P31	64	woman	EGFR.L858R	2	progressive	progressive	–	10	180 genes panel and WGS
P32	66	man	EGFR.L858R	4	stable	stable	Afatinib	3.5	180 genes panel and WGS
P34	60	man	EGFR.L858R	1	progressive	progressive	Gefitinib+Erlotinib	>7	180 genes panel and WGS

LM, leptomeningeal Metastasis; ECOG PS, ECOG performance status; OS, overall survival; TKI, tyrosine kinase inhibitors; CSF, cerebrospinal fluid; WGS, whole genome sequencing.

### Reliable Detection of Driver Mutations in Cerebrospinal Fluid Cell-Free DNA From Leptomeningeal Metastasis Patients

**Figure 1:**
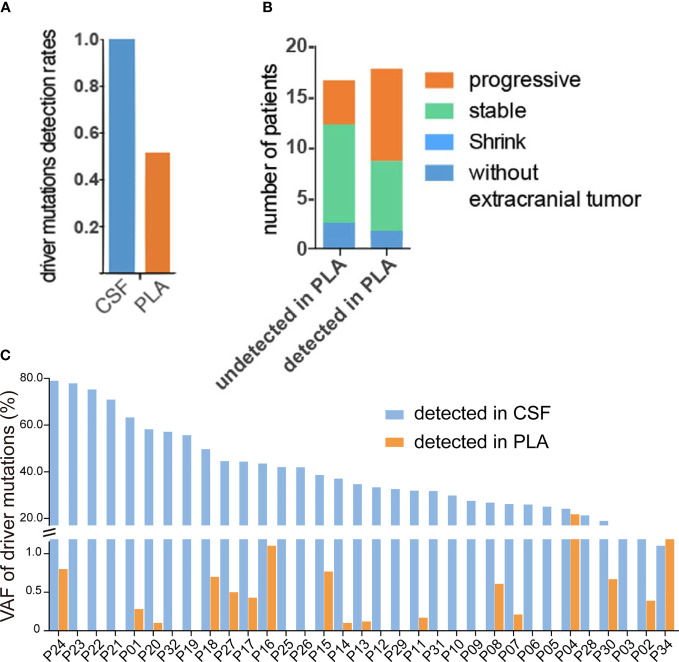
High fraction of ctDNA in CSF. **(A–C)** Targeted sequencing was performed in matched CSF cfDNA and plasma cfDNA, as described in the *Materials and Methods*, and the status of driver mutations was compared in CSF and plasma samples. **(A)** Rates of driver mutation detection in CSF cfDNA and plasma (PLA) cfDNA. **(B)** The association of driver gene mutation detection in PLA cfDNA with extracranial tumor status. The column on the left represents the disease status when driver mutations were not detected and the majority of patients were shown at stable/regression stages. The column on the right represents the disease status when driver mutations were detected and almost 50% of patients were at the progressive stage. **(C)** Variant allele fractions (VAFs) of driver mutations in matched CSF cfDNA or PLA cfDNA samples.

Examination of mutation profiles in CSF cfDNA and matched plasma cfDNA revealed that driver mutations identified in primary tumors could be detected in all CSF cfDNA samples (33 out of 33) but were only present in 51.5% of plasma cfDNA samples (16 out of 33) (**Figure 1A**); these results are in alignment with previous findings that CSF cfDNA is more sensitive than plasma cfDNA in identifying mutations (9, 11–13). The percentage of VAFs was, in general, much higher in CSF cfDNA than in that in plasma cfDNA (**Figure 1C**), with the median VAFs of driver mutations being 34.7% in CSF cfDNA and 0.1% in plasma cfDNA. In addition, 90.9% (30/33) of CSF samples showed VAFs of driver mutations to be over 20%, and only one plasma sample had a VAF of driver mutations of more than 10% (**Figure 1C**). The extremely high VAFs of driver mutations suggested a high fraction of circulating tumor DNA (ctDNA) in CSF cfDNA.

Furthermore, the presence of driver mutations in plasma cfDNA reflected the status of extracranial lesions because patients whose driver mutations were detected in plasma cfDNA were more likely to be at a progressive stage (9 out of 17) and patients whose driver mutations were not detected in plasma were mostly at a stable/regressive stage (**Figure 1B**). For 2 patients who had no extracranial metastases at the time of sample collection, no driver mutations were detected in plasma cfDNA (P12 and P19) (**Table 1** and **Figure 1B**).

### Enrichment of Circulating Tumor DNA in Cerebrospinal Fluid

**Figure 2:**
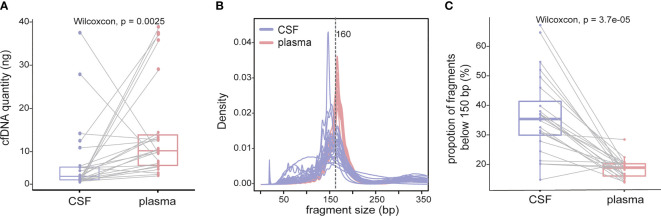
cfDNA fragments are shorter in CSF than those in plasma. cfDNAs were extracted from 24 CSF samples and matched plasma samples, and the size of cfDNA was analyzed by low-coverage WGS (3×). **(A)** cfDNA yield between 5 ml CSF (violet) and 5 ml plasma (red) from 24 NSCLC patients with LM. **(B)** Size distribution of cfDNA fragments between CSF samples and matched plasma samples. **(C)** Proportion of cfDNA fragments below 150 bp in size in CSF samples and matched plasma samples. Each dot represents each sample, and each line connects matching samples.

In plasma cfDNA, ctDNA accounts for a small fraction of cfDNA because most cfDNA is derived from non-cancer cells, especially blood cells (17). It has been reported that cfDNA fragments in the plasma of healthy individuals are significantly longer than those in the plasma of patients with late-stage lung cancer, and therefore, selective sequencing with specific fragment sizes may boost ctDNA detection (17). In a parallel comparison, the yield of cfDNA from CSF was much lower than that from plasma (median yield: 1.75 ng/ml of CSF vs. 8.19 ng/ml of plasma, Wilcoxon test p value = 0.0025) (**Figure 2A**); 53.8% of CSF cfDNA samples showed a size of peak fragments below 160 bp, while the size of peak fragments from all plasma cfDNA samples was over 160 bp (**Figure 2B**). Consistent with this, the proportion of CSF cfDNA in the size range of 20–150 bp was significantly higher than that for plasma cfDNA (**Figure 2C**). A reduction in cfDNA fragment size and extremely high VAFs of driver mutations in CSF provided evidence that ctDNA was more enriched in CSF and better represents LM-associated genetic alterations.

### Leptomeningeal Metastases and Extracranial Lesions Acquired Resistance Mutations Independently

**Figure 3:**
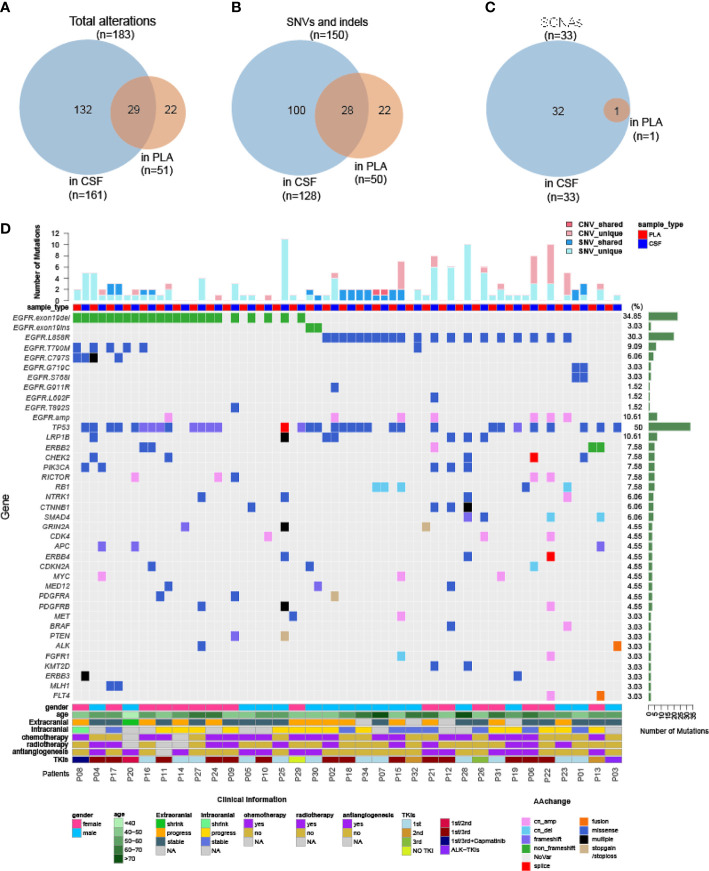
Genomic landscape of CSF and plasma cfDNA in NSCLC with LM. Samples were subject to targeted sequencing by different vendors; a final analysis for 84 core genes was performed, and the results were compared between CSF and plasma. **(A–C)** Venn diagram of genetic alterations for CSF- and plasma-based cfDNA profiling. **(A)** Venn diagram of total alterations detected in CSF cfDNA and plasma (PLA) cfDNA. **(B)** Venn diagram of single-nucleotide variants (SNVs) and indels detected in CSF cfDNA and PLA cfDNA. **(C)** Venn diagram of somatic copy number alterations (SCNAs) detected in CSF cfDNA and PLA cfDNA. **(D)** Mutational landscape in plasma cfDNA and matched CSF cfDNA from individual patients. The number of SNVs and SCNAs are shown at the top of the panel, and clinical information is shown at the bottom of the panel.

As demonstrated by driver mutation detection, plasma cfDNA was not reflective of LM status (**Figure 1**). This finding was further confirmed when comparing more comprehensive genomic profiles between CSF cfDNA and plasma cfDNA. Among all 183 alterations detected in all samples by targeted sequencing, only 29 were present in both CSF cfDNA and matched plasma cfDNA samples (**Figure 3A**, cutoff limit: Mutant allele frequency (MAF) >0.1%), including 28 single-nucleotide variants (SNVs) or indels and 1 CNV (**Figures 2B, C**); 132 were unique to CSF (not present in matched plasma), and 22 mutations were unique to plasma (not present in matched CSF) (**Figure 3A**). The presence of plasma-specific genetic alterations was more striking when considering the high sensitivity of CSF in picking up mutations for LM, suggesting the possibility that CSF cfDNA and plasma cfDNA can be used to detect separate tumors located in different compartments with distinct features.

**Figure 4:**
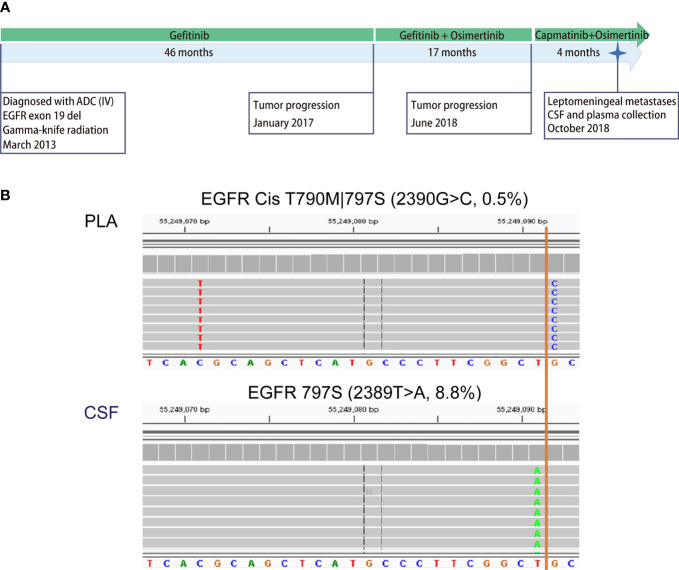
Case analysis: distribution of EGFR-TKI-resistant mutations in P08. Patient P08 was exposed to both first- (gefitinib) and third-generation (osimertinib) EGFR-TKIs before LM diagnosis. **(A)** Disease timeline. P08 was diagnosed with lung adenocarcinoma in March 2013 and received treatment with gefitinib, osimertinib, and capmatinib sequentially. In October 2018, he was diagnosed with leptomeningeal metastasis. CSF and matched peripheral blood were collected subsequently. **(B)** The mutation profiling of CSF cfDNA and plasma cfDNA was performed using a next-generation sequencing (NGS) panel containing 180 genes. The raw reads for two types of EGFR mutations are shown. EGFR C797S 2390G>C was detected in the CSF in cis with T790M 2369C>T in plasma only (0.6%), not in CSF, while C797S 2389T>A was detected in CSF only (8.8%).

The large number of CSF-specific mutations, which could not be detected in plasma, might be simply a result of the low sensitivity of mutation detection *via* plasma biopsy. Plasma-specific mutations reveal differences between LM and extracranial lesions. Therefore, we focused on 22 plasma-specific mutations. The EGFR T790M mutation, the most common acquired resistant mutation to first-/second-generation EGFR-TKIs with poor blood–brain barrier (BBB) penetration, was the most frequently detected plasma-specific mutation in this cohort. When tracking the treatment history of these patients, among 28 patients who were treated with first-/second-generation EGFR-TKIs, the EGFR T790M mutation was detected in 5 plasma cfDNA samples (**Figure 3D**; P04, P08, P16, P17, and P20) and in one CSF cfDNA sample (**Figure 3D**, P32). The lack of effective exposure of meningeal metastases to first-/second-generation EGFR-TKIs (23) may be one of the reasons for the lesser detection of EGFR T790M mutations in CSF. However, the EGFR C797S mutation was almost evenly distributed in CSF and matched plasma collected from third-generation EGFR-TKI osimertinib-treated patients (n = 15). The incidence of EGFR T790M in plasma and CSF was not significantly different due to the small sample size. Notably, however, the EGFR C797S mutation was detected in both CSF and plasma, but with different nucleotide variants in patient P08. In plasma, EGFR 2390G>C was located in cis to T790M (VAF: 0.5%). But in the CSF, it was replaced by an EGFR 2389T>A (VAF: 8.8%) (**Figures 4A, B**). In other words, LM and extracranial lesions independently acquired resistance mutations in patient P08. In our cohort, EGFR-TKI-resistant mutations with the same nucleotide variant were never present in both CSF and plasma from the same patient.

These results suggest that EGFR-TKI-resistant mutations evolved differently in the extracranial lesion and LM from the same patient, providing further evidence that extracranial and LM lesions progress independently and supporting the notion that both CSF cfDNA and plasma cfDNA are necessary for comprehensive genetic profiling to make clinical decisions for NSCLC patients with LM.

### Chromosomal Instability as a Universal Genetic Characteristic of Leptomeningeal Metastasis

Given the evidence that CSF cfDNA is representative of genetic profiles for LM lesions, we further characterized LM by CSF cfDNA. It is worth noting that in 7 CSF samples (22.6%), VAFs of EGFR mutations were over 50% (**Figure 1C**), indicating a loss of heterozygosity (LOH) of EGFR in LM. Similarly, almost all CNVs were exclusively detected in CSF samples in this cohort (**Figures 3C, D**). The high occurrence rate of both LOH and CNVs in CSF cfDNA suggested a universal genome instability in LM.

**Figure 5:**
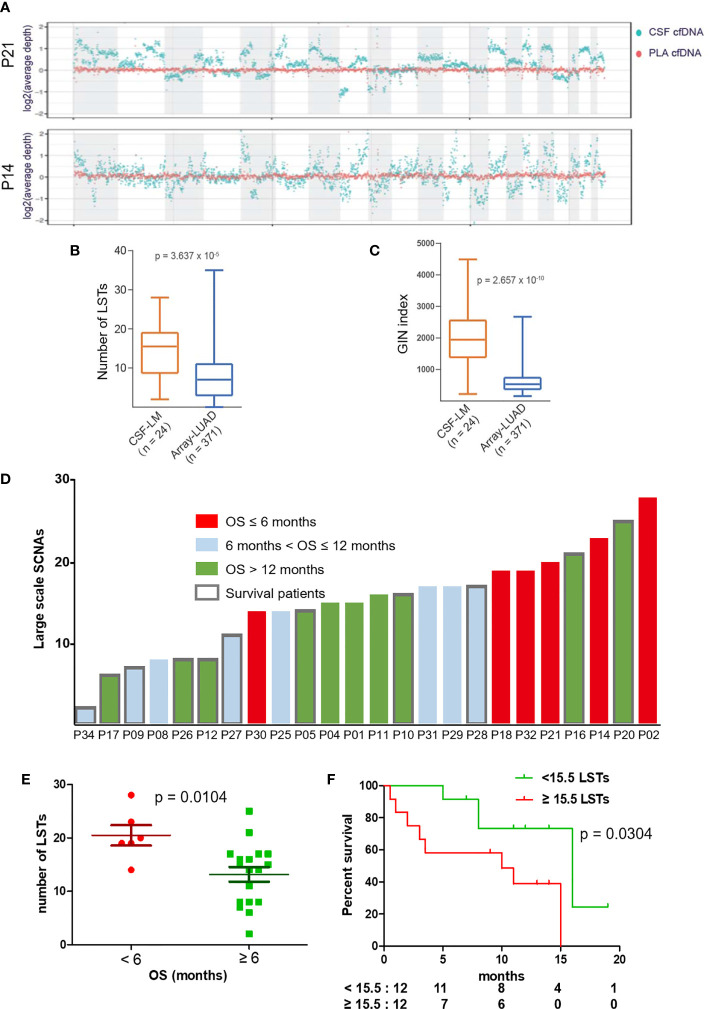
LST as a potential prognostic marker for LM. **(A)** Visualization of genomic instability. The average depths along chromosomes (1M/bin) of CSF cfDNA (teal color) and matched plasma cfDNA (orange color) from 2 representative patients are shown. Plasma samples demonstrated a constant read across the chromosome, while reads from CSF samples varied continuously, indicating extensively affected copy numbers through the whole genome. **(B, C)** Genomic instability was assessed based on the number of large stable transitions [large-scale state transitions (LSTs)] and genome instability number (GIN) using low-coverage WGS data for the current cohort (n = 24, CSF-LM) and compared with a published dataset comprising genomic DNA profiles from primary lung adenocarcinoma tissues [n = 371, Array-LUAD (24)]. Data were analyzed as described in the *Materials and Methods*. **(B)** Comparison of LST between CSF-LM and Array-LUAD. The average level of LSTs in CSF-LM was significantly higher than that in Array-LUAD (15.0 vs. 7.8, p < 0.001). **(C)** Comparison of GIN levels between CSF-LM and Array-LUAD. The average level of GIN in CSF-LM was significantly higher than that from Array-LUAD (1,961.4 vs. 681.6, p < 0.001). **(D)** Levels of LSTs and survival status in LM patients (n = 24). Each column represented each patient. Columns in red indicate an overall survival of less than 6 months, columns in green indicate an overall survival of over 12 months, columns in gray indicate an overall survival between 6 and 12 months. The columns with borders indicate the patients who were still alive at the time of the data collection and reporting, while the columns without borders indicate patients who had died at the time of data collection. **(E, F)** Association of LSTs with survival. **(E)** The levels of LSTs were significantly lower in patients whose survival was over 6 months (p = 0.0068). **(F)** Kaplan–Meier curves of overall survival. With a cutoff at 15.5, the median level of this cohort, patients with higher levels of LSTs demonstrated a short survival (median OS after LM, 10 months vs. 15 months, p = 0.038). However, GIN was not associated with survival (**Figure S1**).

To assess genome instability, we performed low-coverage WGS (3×) with matched CSF and plasma cfDNA samples from 24 out of 33 patients. At the whole-genome level, CSF cfDNA demonstrated a much more vibrating pattern (**Figure 5A**). LSTs (longer than 10 Mb) were detected in 23 CSF cfDNA samples (95.8%, 23/24, median number of LSTs is 15.5) compared with only 2 plasma cfDNAs (8.3%, 2/24). The observation of high levels of genomic instability in LM was further confirmed by comparing to primary lung adenocarcinoma tissue samples from a public dataset (24) (**Figures 5B, C**, labeled as LUAD). Both the number of LST and GIN of this cohort were significantly higher in CSF cfDNA than in genomic DNA derived from primary tumor tissues (**Figures 5B, C**).

### Association of Large-Scale State Transitions in Cerebrospinal Fluid With Patient Survival

To further understand the relationship between chromosomal instability and the clinical outcome of LM patients, we analyzed data from 24 patients with a follow-up over 6 months (**Figure 5D**) and found that a survival shorter than 6 months was correlated with higher levels of LSTs (**Figure 5E**). In contrast, 6-month survival rates in patients with higher levels of LSTs (≥15.5) and lower levels of LSTs (<15.5) were 60% and 92%, respectively. Median survival was significantly shorter in patients with higher levels of LSTs (10 months vs. 16 months, p = 0.0304, **Figure 5F**). GIN levels were not found to be associated with patient survival (**Figure S1**).

## Discussion

The rate of detection of mutations in CSF from patients is lower when tumors are located farther away from the cerebral ventricle (25, 26). However, LM is adjacent to CSF, and this study and others reveal that it shows 100% sensitivity in identifying driver mutations through CSF-based biopsy (**Figure 1A**). Compared with plasma cfDNA, the enrichment of ctDNA in CSF could explain its high sensitivity. LMs are normally diagnosed through MRI or CSF cytology with low sensitivity before patients receive lifesaving treatments (5, 6, 27). CSF cfDNA, which contains a high fraction of ctDNA, has shown great promise in diagnosing LM over traditional methods with its high sensitivity as a result of the enrichment of LM-specific mutations. In addition, we found that genome instability is a universal genetic characteristic of LM. CSF cfDNA-based low-coverage WGS might offer an early and sensitive diagnostic tool in LM, especially for patients without hotspot mutations. CSF-based dynamic mutation profiling and low-coverage WGS, in combination with MRI or CSF cytology, could be set up conveniently in a hospital setting to offer an early diagnosis and accurate assessment of disease status.

The difference in detection sensitivity makes the direct comparison between CSF and plasma difficult. Under most conditions, the MAF of a particular genetic change in CSF is 50- to100-fold higher than that in plasma (**Figure 1**). Therefore, it is not surprising that CSF-specific alterations dramatically outnumber plasma-specific alterations (**Figure 3**). At the same time, the difference in detection sensitivity makes plasma-specific alterations more clearly evident. The predominant presence of T790M in plasma cfDNA can be explained by poor BBB penetration of first-/second-generation EGFR-TKIs (23). However, the different nucleotide mutations of C797S detected in CSF and plasma, i.e., 2390G>C only in plasma and 2389T>A only in CSF, from one patient upon treatment with improved BBB-penetrating osimertinib support the independent evolution of extracranial lesions and LMs. In addition to resistance mutations, very few passenger mutations (**Figure 3D**, such as GRIN24 in P21 and CDKN24 in P30) were detected exclusively in plasma, and the mechanism by which tumors in these two compartments behave differently remains unclear. Resolution of this discrepancy requires larger cohort studies.

The potential biomarker LST identified in LM through CSF-based liquid biopsy in this study offers new possibilities for understanding and potentially exploring novel treatment strategies for LM. However, the cutoff value for LST needs to be determined with a larger dataset. Despite the fact that both GIN and LST are considered indicators of chromosomal ploidy, instead of considering all CNVs in GIN index evaluation (22), LSTs only count large-scale CNVs that are longer than 10 Mb (21). LSTs are reported as indicators of homologous recombination deficiency (HRD) and associated with cisplatin and poly ADP ribose polymerase (PARP) inhibitor sensitivity in breast carcinomas (28). This might explain why both LST and GIN were higher in LMs, but only LST was associated with survival (**Figure 5**, **Figure S1**). The findings are expected to guide future investigations of HRD in LM for new therapeutic opportunities (28).

In summary, our study confirms previous findings that CSF is a more sensitive and reliable liquid biopsy tool for LM, suggesting that LM and extracranial lesions arise through independent processes during cancer development and identifying higher levels of LSTs in CSF as a prognostic marker. Based on findings from this study, a simple driver mutation test and low-coverage WGS through CSF biopsy might offer a ready-to-use diagnostic tool for LM. Future larger-scale studies on HRD might lead to new opportunities for the treatment of LM. The discrepancy between extracranial lesion(s) and LM supports the evaluation of the disease *via* a combination-based approach for a more accurate assessment of disease status and clinical intervention.

## Data Availability Statement

The datasets presented in this study can be found in online repositories. The name of the repository and accession number can be found below: Bio-Med Big Data Center (BMDC) National Omics Data Encyclopedia (NODE), https://www.biosino.org/node/, OEP003149.

## Ethics Statement

The studies involving human participants were reviewed and approved by The Independent Ethics Committee, National GCP Center for Anticancer Drugs, China. The patients/participants provided their written informed consent to participate in this study.

## Author Contributions

PX and JL designed and supervised the study. XW and MS xsearched the literature. XW, PX, JX, and JW participated in data acquisition. MS, WG, FZ, and HZ did the data analysis and interpretation. XW and MS drafted the article. WG, FZ, and HZ did statistical analysis. PX and JL performed critical revision of the article for important intellectual content. All authors contributed to the article and approved the submitted version.

## Conflict of Interest

Authors MS, WG, FZ, and HZ were employed by Hangzhou Jichenjunchuang Medical Laboratory Co., Ltd.

The remaining authors declare that the research was conducted in the absence of any commercial or financial relationships that could be construed as a potential conflict of interest.

## Publisher’s Note

All claims expressed in this article are solely those of the authors and do not necessarily represent those of their affiliated organizations, or those of the publisher, the editors and the reviewers. Any product that may be evaluated in this article, or claim that may be made by its manufacturer, is not guaranteed or endorsed by the publisher.
