# Supplementary material for: Cerebrospinal Fluid Cell-Free DNA-Based Detection of High Level of Genomic Instability Is Associated With Poor Prognosis in NSCLC Patients With Leptomeningeal Metastases
Source: Front Oncol. 2022 Apr 28;12:664420. doi: 10.3389/fonc.2022.664420 (PMC9097599; doi:10.3389/fonc.2022.664420)

Table S1 84 genes for targeted sequencing analysis.

Figure S1. GIN index in CSF cfDNA was not associated with prognosis in NSCLC patients with LM. **(A)** Level of GIN index was comparable between patients with an OS of more than 6 months and less than 6 months (mean GIN 2385.5 vs. 1820.2, p=0.1979). **(B),** Kaplan–Meier curves of overall survival. With a cutoff at 1944, the median level of this cohort, patients with higher levels of GIN had a similar OS as the patients with lower levels of GIN (median OS after LM, 9 months vs 15 months, p=0.77).


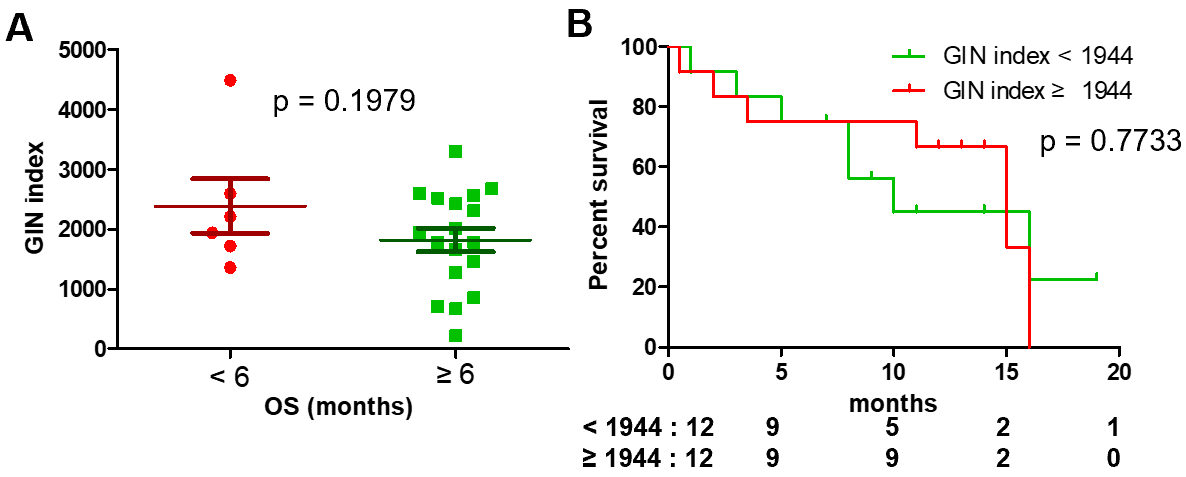

Supplement: Supplementary file 1 [file DataSheet_1.docx]
